# Comparative transcriptomic analysis of antimony resistant and susceptible *Leishmania infantum* lines

**DOI:** 10.1186/s13071-020-04486-4

**Published:** 2020-11-30

**Authors:** Juvana Moreira Andrade, Leilane Oliveira Gonçalves, Daniel Barbosa Liarte, Davi Alvarenga Lima, Frederico Gonçalves Guimarães, Daniela de Melo Resende, Ana Maria Murta Santi, Luciana Marcia de Oliveira, João Paulo Linhares Velloso, Renato Guimarães Delfino, Pascale Pescher, Gerald F. Späth, Jeronimo Conceição Ruiz, Silvane Maria Fonseca Murta

**Affiliations:** 1 Genômica Funcional de Parasitos, Instituto René Rachou, Fiocruz Minas, Belo Horizonte, MG Brazil; 2 Grupo Informática de Biossistemas, Instituto René Rachou, Fiocruz Minas, Belo Horizonte, MG Brazil; 3 Programa de Pós-graduação em Biologia Computacional e Sistemas, Instituto Oswaldo Cruz, Fiocruz, Rio de Janeiro, RJ Brazil; 4 Universidade Federal do Piauí, Teresina, PI Brazil; 5 Unité Biologie des ARN des Pathogènes Fongiques, Département de Mycologie, Institut Pasteur, Paris, France; 6 Unité de Parasitologie moléculaire et Signalisation, Département de Parasitologie et Mycologie, Institut Pasteur, Paris, France

**Keywords:** *Leishmania infantum*, Trivalent antimony, Resistance, RNA sequencing, Transcriptome, Differentially expressed genes

## Abstract

**Background:**

One of the major challenges to leishmaniasis treatment is the emergence of parasites resistant to antimony. To study differentially expressed genes associated with drug resistance, we performed a comparative transcriptomic analysis between wild-type and potassium antimonyl tartrate (Sb^III^)-resistant *Leishmania infantum* lines using high-throughput RNA sequencing.

**Methods:**

All the cDNA libraries were constructed from promastigote forms of each line, sequenced and analyzed using STAR for mapping the reads against the reference genome (*L. infantum* JPCM5) and DESeq2 for differential expression statistical analyses. All the genes were functionally annotated using sequence similarity search.

**Results:**

The analytical pipeline considering an adjusted *p*-value < 0.05 and fold change > 2.0 identified 933 transcripts differentially expressed (DE) between wild-type and Sb^III^-resistant *L. infantum* lines. Out of 933 DE transcripts, 504 presented functional annotation and 429 were assigned as hypothetical proteins. A total of 837 transcripts were upregulated and 96 were downregulated in the Sb^III^-resistant *L. infantum* line. Using this DE dataset, the proteins were further grouped in functional classes according to the gene ontology database. The functional enrichment analysis for biological processes showed that the upregulated transcripts in the Sb^III^-resistant line are associated with protein phosphorylation, microtubule-based movement, ubiquitination, host–parasite interaction, cellular process and other categories. The downregulated transcripts in the Sb^III^-resistant line are assigned in the GO categories: ribonucleoprotein complex, ribosome biogenesis, rRNA processing, nucleosome assembly and translation.

**Conclusions:**

The transcriptomic profile of *L. infantum* showed a robust set of genes from different metabolic pathways associated with the antimony resistance phenotype in this parasite. Our results address the complex and multifactorial antimony resistance mechanisms in *Leishmania*, identifying several candidate genes that may be further evaluated as molecular targets for chemotherapy of leishmaniasis.
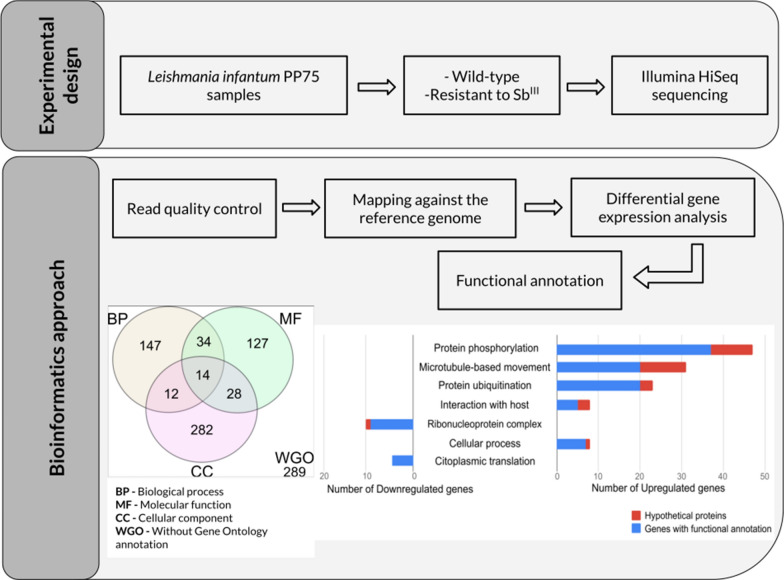

## Background

Leishmaniasis is a complex of diseases caused by different species of the protozoan parasite *Leishmania* (Kinetoplastida, Trypanosomatidae) that represents one of the major public health problems in developing countries according to the World Health Organization [1]. Human leishmaniasis has a prevalence of 12 million cases and an incidence of 0.7–1.0 million new cases annually from nearly 100 endemic countries, with an estimated population of more than 1 billion people at risk of infection [1, 2]. Depending on genetic and environmental factors, the host immune response and mainly on the *Leishmania* species involved, the disease can comprise two main clinical forms: visceral or cutaneous leishmaniasis [3]. Visceral leishmaniasis (VL)—caused by *Leishmania *(*Leishmania*)* donovani* in Asia and Africa and *Leishmania *(*Leishmania*)* infantum* (syn. *L. *(*L.*)* chagasi*) in the Mediterranean Basin, the Middle East, Central Asia, South America and Central America—is the most severe, systemic form and is lethal if not treated [3, 4]. The estimated incidence of VL is approximately 50,000 to 90,000 cases per year, and this disease remains endemic in more than 60 countries [1]. More than 95% of global VL cases occur in ten countries: Brazil, China, Ethiopia, India, Iraq, Kenya, Nepal, Somalia, South Sudan and Sudan [1].

There is no human vaccine available against *Leishmania* infections, and the control is based mainly on chemotherapy. Pentavalent antimony-containing compounds such as sodium stibogluconate (Pentostam®) and *N*-methyl-glucamine (Glucantime®) have been used as the first-line therapies against all forms of leishmaniasis. Although antimony's action has not been fully elucidated, studies suggest that pentavalent antimony (Sb^V^) is reduced in vivo to the trivalent active form (Sb^III^) [5]. Literature data have indicated that antimony inhibits macromolecule biosynthesis in amastigotes, possibly via inhibition of glycolysis and fatty acid oxidation [6]. An earlier report indicated that antimonials cause perturbations in the thiol redox potential, driving to parasite death by oxidative stress [7]. Other studies have shown that antimony causes DNA fragmentation and can kill the parasite by an apoptotic process [8, 9]. In addition, zinc finger proteins have also been recognized as potential targets of Sb^III^ [10].

One major challenge for leishmaniasis treatment is the emergence of parasites resistant to Sb^V^ [11, 12]. Treatment failure with Sb^V^ has been reported in Bihar (India), where more than 60% of patients with VL are unresponsive to Sb^V^ [4, 13]. Different mechanisms of drug resistance have been reported [11], including decreased Sb^III^ entry into the cell due to reduced expression of aquaglyceroporin (AQP1) [14–16] or sequestration of the metal–thiol conjugate into vesicular membranes of *Leishmania* by the ATP-binding cassette transporter [17, 18] and greater Sb^III^ efflux due to amplification of ABC transporters [19]**.**

Decuypere et al. [20] showed that the molecular changes associated with antimonial resistance in *Leishmania* isolates depend on their genetic background. To understand the mechanisms responsible for drug resistance in *Leishmania*, different approaches have been used. These include gene expression analyses of antimony-resistant *L. amazonensis* by DNA microarrays [21], proteomic analyses of Sb^III^-resistant *L. braziliensis* and *L. infantum* [22] and phosphoproteomic analysis of Sb^III^-resistant and -susceptible *L. braziliensis* [23].

Several studies showed the use of next-generation sequencing technologies to contribute to a better understanding of *Leishmania* biology. These have been widely used for comparing the gene expression profiles of primary cutaneous lesions from patients infected with *L. braziliensis* [24], to analyze the global changes in gene expression during *L. major* differentiation from procyclic to metacyclic forms [25] and for an overview of the global transcriptome of the *L. major* promastigote stage [26]. Recently, transcriptomic changes in an in vitro-adapted *L. amazonensis* in response to Sb^III^ and comparative genomic and transcriptome analysis of Sb^III^-resistant and -susceptible *L. braziliensis* and *L. panamensis* were performed using DNA and RNA sequencing [27, 28]. To the best of our knowledge, the transcriptomic analysis associated with antimony resistance in *L. infantum* has not yet been addressed. Thus, this study attempts to perform a comparative transcriptomic analysis (RNA-seq) between Sb^III^-resistant and wild-type *L. infantum* lines.

## Methods

### *Leishmania* samples

This study used lines of *L. infantum* (MHOM/BR/74/PP75) wild type (LiWTS) and resistant to potassium antimonyl tartrate (Sb^III^) (LiSbR). The resistant line (LiSbR) was previously selected in vitro by a step-wise increase of Sb^III^ drug pressure [29]. These parasites were further maintained in culture under Sb^III^ pressure, and the effective concentration required to decrease growth by 50% (EC_50_) was determined using a model Z1 Coulter Counter (Beckman Coulter, Fullerton, CA, USA). EC_50_ values for LiWTS and LiSbR obtained in this study were 0.12 mg/ml and 1 mg/ml, respectively, with an eight-fold resistance index (data not shown). Then, promastigote forms of these lines were grown at 26 °C in M199 medium supplemented with 40 mM HEPES pH 7.4, 1 μg/ml biotin, 5 μg/ml hemin, 2 μg/ml biopterin, 2 mM l-glutamine, 500 U penicillin, 50 μg/ml streptomycin and 10% (v/v) heat-inactivated fetal bovine serum [29]. Three independent biological replicates of each line were cultured. Based on previous studies of our group [29], wild-type *L. infantum* parasites were incubated for 24 h in the absence of drug (LiWTS 0), and resistant parasites were treated with 0.06 mg/ml Sb^III^ (LiSbR 0.06), which corresponds to half of the Sb^III^ IC_50_ for the LiWTS line. Cells were washed in RPMI medium, pelleted by centrifugation and frozen at − 70 °C.

### RNA-Seq library preparation and sequencing

Promastigote forms were harvested, lysed and homogenized in the presence of guanidine-thiocyanate-containing buffer, and total RNA was extracted using the RNA extraction kit (RNeasy-QIAGEN, Valencia, CA, USA), according to the manufacturer’s instructions. After extraction, total RNA was analyzed on the Agilent Bioanalyzer (Santa Clara, CA, USA) for quality and integrity assessment and, after this, submitted to cDNA synthesis. All samples presented an RNA integrity number (RIN) ≥ 6.8.

The construction of six non-directional libraries was prepared using the TruSeq RNA Sample preparation v2 protocol (Illumina, Inc., San Diego, CA), using 5 µg of total RNA for each library. The Illumina HiSeq2000 technology (Illumina, Inc., San Diego, CA) of Sequencing Platform of the Institut Pasteur was used to sequence the samples, based on directional sequencing of 100-bp-long reads of retro-transcribed mRNAs.

### Genome data used

*Leishmania infantum* JPCM5 genome data were downloaded from *European Nucleotide Archive* (ENA; http://www.ebi.ac.uk/ena/) under accession number ena-STUDY-CBMSO-04-04-2017 [30]. This genome version refers to the resequencing of the *L. infantum* JPCM5 genome at the end of 2017. According to Fuente et al. [30], 495 new genes have been annotated, 100 have been corrected, and 75 previous annotated genes have been discontinued. The TriTrypDB version contains a chromosome LinJ.00 formed by 34 genomic regions of uncertain chromosomal location, and in the new genome version all regions were identified in the correct chromosomal location.

### Data quality control

Raw sequence reads in FASTQ format were evaluated in terms of read quality (per base sequence quality, per base G+C content, sequence length distribution, sequence duplication levels, Kmer content and low complexity sequences) with PRINSEQ [31]. Data filtering and trimming were performed with Trimmomatic [32]. Sequence artifacts such as sequencing adapters were removed using data available in the Trimmomatic software package.

One cDNA library from the LiSbR line sequenced was removed from RNA seq analysis, since it showed a low throughput and small coverage (< 60×) compared to the other two libraries from the same *L. infantum*-resistant line (approximately 200×).

A curated General Feature Format (GFF) file was generated from the updated genome annotation and used to guide the alignment process. Reads were aligned in the reference genome with STAR [33], allowing up to three mismatches per read.

Mapped reads were converted to SAM format with SAMtools [34] and visualized with the Integrative Genome Viewer (IGV) [35].

### Differential expression analysis

To perform the differential gene expression analysis, HTSeq-count [36] was used to count the total number of mapped reads for each annotated gene in the GFF file. For differential gene expression discovery, DESeq2 [37] was used. To identify differentially expressed (DE) genes, an adjusted *p*-value < 0.05 and fold-change (FC) > 2.0 were set as thresholds to define the significance.

### Functional analysis

Blast2GO [38, 39] version 5.1.13 was used to map the DE genes in the gene ontology (GO) database [40]. The functional enrichment analysis (Fisher’s exact test) was performed in the Blast2GO software using as test set the lists of DE genes and as reference the *L. infantum* JPCM5 predicted proteome. A false discovery rate (FDR) and an adjusted *p*-value < 0.05 were set as thresholds to define the functional enrichment significance.

## Results

### Overview of samples sequencing

The purpose of this study was to compare the transcriptome of Sb^III^-resistant (LiSbR) and wild-type (LiWTS) *L. infantum* lines. For this, cDNA libraries from these samples were constructed, sequenced and analyzed, allowing the identification of differential gene expression associated with Sb^III^ resistance mechanisms.

Three independent biological replicates of the wild-type parasites and two of the Sb^III^-resistant parasites were sequenced, producing ~ 500 million 100 base pair reads. After quality trimming (adaptor removal and Phred quality cutoff ≥ 25), approximately 2% of the reads were lost. After mapping, approximately 388 million reads were aligned to a reference genome (*L. infantum* JPCM5).

### Differential expression analysis

In the dataset comprising 8591 protein coding transcripts (obtained from ENA database), 933 (933/8591, 10.86%) DE transcripts were identified between wild-type and Sb^III^-resistant *L. infantum* lines considering the applied cutoffs of adjusted *p*-value < 0.05 and FC > 2.0. Out of 933 DE transcripts, 504 (504/933, 54.01%) presented functional annotation and 429 (429/933, 45.99%) were assigned as hypothetical proteins without predicted function (Table 1). A total of 837 (837/933, 89.71%) transcripts were upregulated and 96 (96/933, 10.29%) were downregulated in the Sb^III^-resistant *L. infantum* (Table 1).

**Table 1:** Transcripts differentially expressed between wild-type and Sb^III^-resistant *L. infantum* lines and Gene Ontology (GO) functional enrichment analysis

	GO category: biological process		GO category: cellular component or molecular function	Without GO	Total
	Enriched	Not enriched			
Functional annotation^a^					
Upregulated	111 (11.90%)	13 (1.39%)	289 (31.94%)	18 (1.93%)	431 (46.19%)
Downregulated	37 (3.96%)	0	6 (0.64%)	30 (3.21%)	73 (7.82%)
Hypothetical proteins^b^					
Upregulated	32 (3.43%)	13 (1.39%)	142 (15.22%)	219 (23.47%)	406 (43.51%)
Downregulated	1 (0.11%)	0	0	22 (2.36%)	23 (2.79%)
Total number of DE transcripts identified	181	26	437	289	933

^a^Transcripts with functional annotation

^b^Transcripts with no assigned function

The functional enrichment analysis of 933 transcripts obtained in this study was performed on Blast2GO software [38, 39]. Out of 933 transcripts, 644 (644/933, 69.02%) were associated with some GO ontology related to the biological process (BP), molecular function (MF) or cellular component (CC) and 289 (298/933, 30.98%) did not present any associated GO term (Table 1). A total of 207 (207/644, 32.14%) DE transcripts presented GO ontology on biological processes, 181 (181/207, 87.44%) being functionally enriched, and 26 (26/207, 12.56%) did not show enrichment (Table 1).

**Fig. 1:**
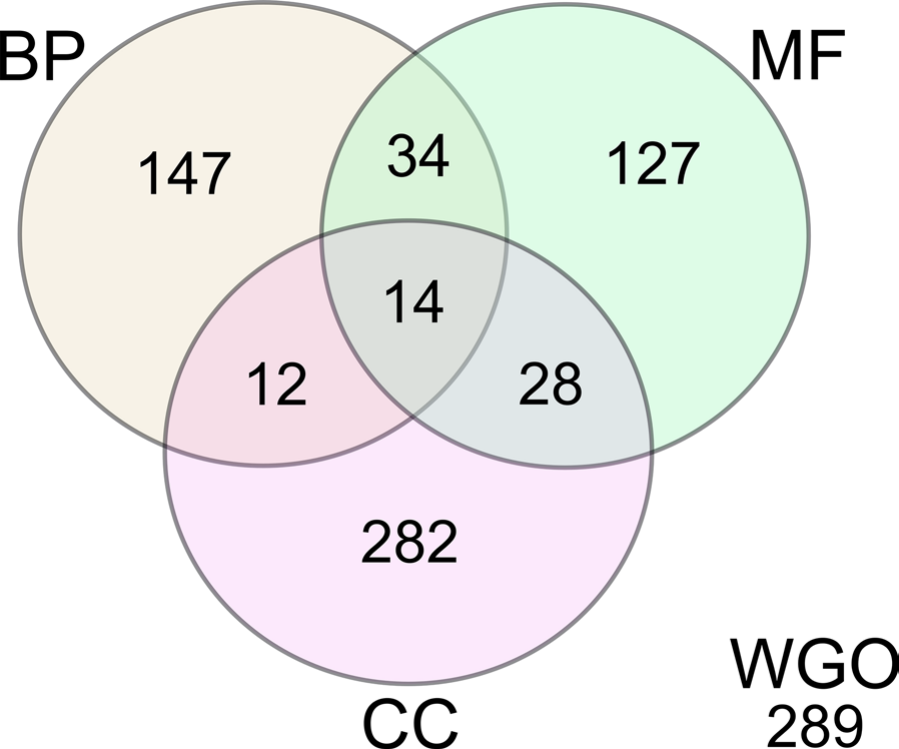
Venn diagram of shared and specific Gene Ontology terms for the differentially expressed transcripts. The 644 differentially expressed genes (FC ≥ 2) of antimony-resistant *L. infantum* were compared and grouped together using the Gene Ontology categories (*BP* biological process, *MF* molecular function, *CC* cellular component). The total amount of shared and specific sequences in each ontology group is depicted in the figure. In addition, 289 differentially expressed genes (FC ≥ 2) were not assigned to any gene ontology category (WGO)

The distribution of 644 differentially expressed transcripts in the three different GO categories, BP, MF and CC, is represented in the Venn diagram (Fig. 1).

Many transcripts are associated with more than one GO ontology. The majority of transcripts correspond to cellular components (282/644, 47.79%), followed by biological processes (147/644, 22.83%) and molecular function (127/644, 19.72%). Fourteen transcripts present all three categories.

**Fig. 2:**
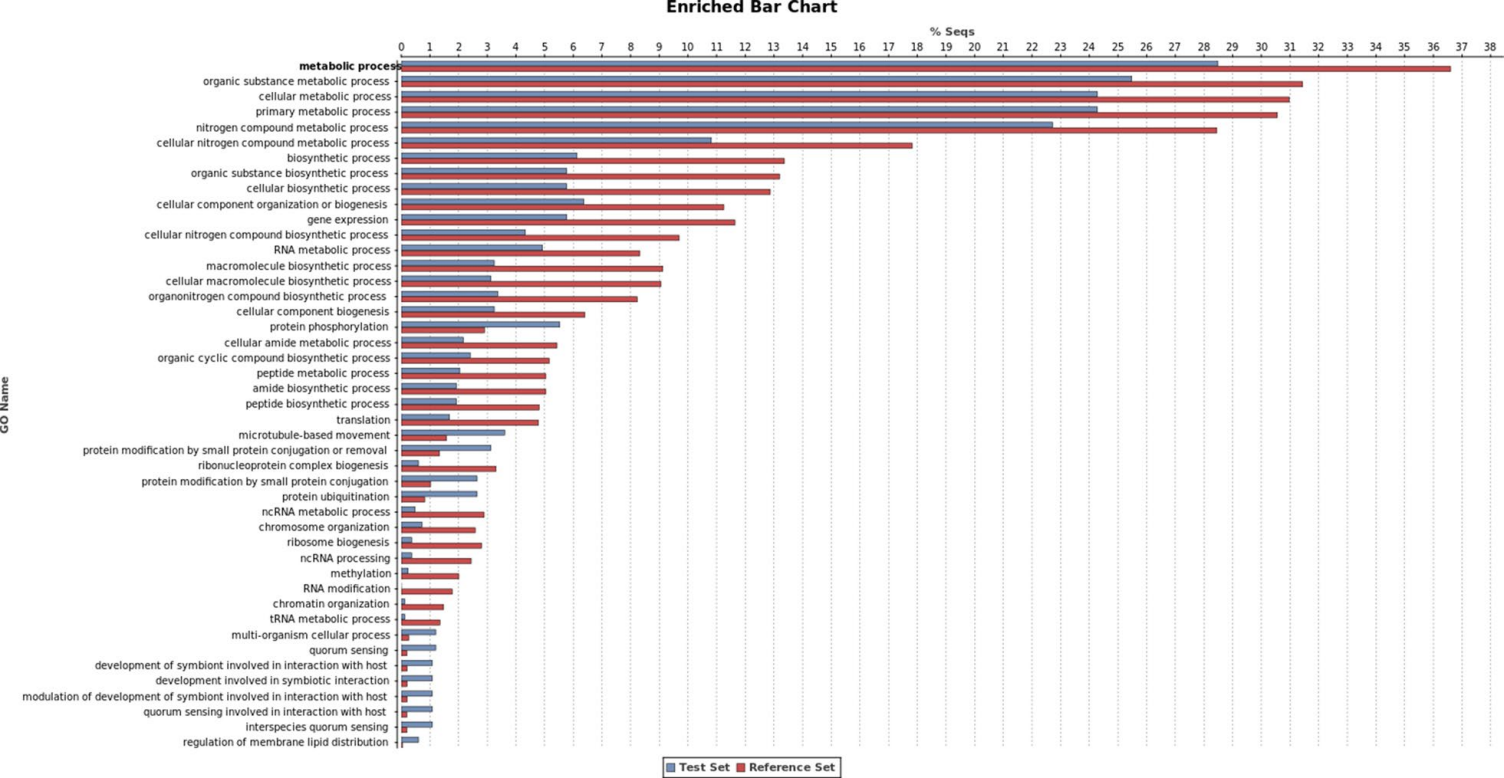
Gene Ontology enrichment analysis for the upregulated transcripts. The GO enrichment analysis (Fisher’s exact test) was performed using as test set the list of upregulated transcripts and as reference set the *L. infantum* JPCM5 predicted proteome. FDR < 0.05 was set as a threshold to define the functional enrichment significance. The percentage of sequences in each GO category is described in the *Y* axis. Red bars represent the percentage of sequences classified in each GO term for the “reference set” group, and the blue bars represent the percentage of sequences classified in each GO term for the “test set” group (DE genes)

**Fig. 3:**
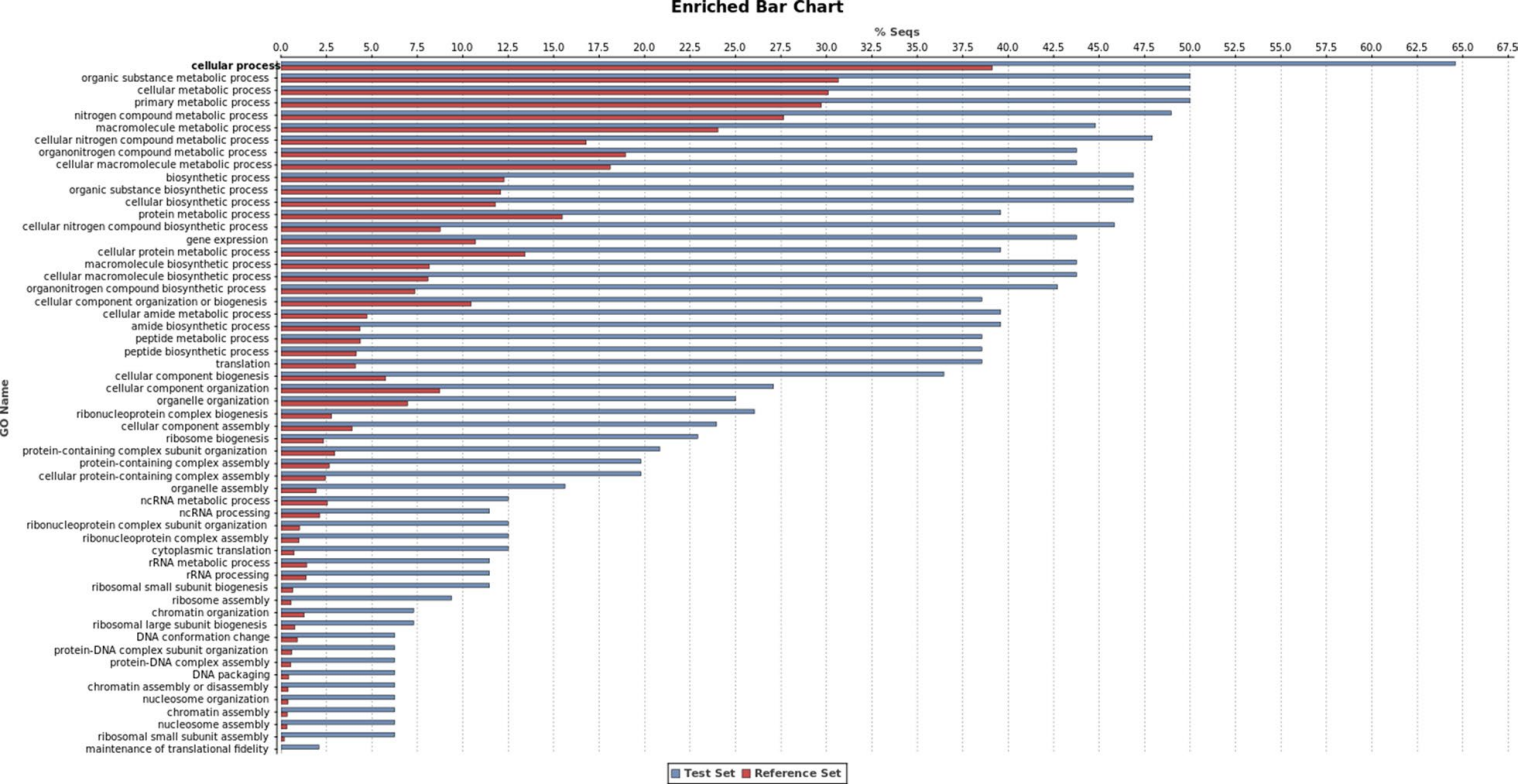
Gene Ontology enrichment analysis for the downregulated transcripts. The GO enrichment analysis (Fisher’s exact test) was performed using as test set the list of downregulated transcripts and as reference set the *L. infantum* JPCM5 predicted proteome. FDR < 0.05 was set as a threshold to define the functional enrichment significance. The percentage of sequences in each GO category is described in the *Y* axis. Red bars represent the percentage of sequences classified in each GO term for the “reference set” group, and the blue bars represent the percentage of sequences classified in each GO term for the “test set” group (DE genes)

Gene ontology enrichment analysis (Fisher’s exact test) was performed using as “test set” the list of upregulated (Fig. 2) and downregulated (Fig. 3) differentially expressed transcripts (DE) and as “reference set” (background) the *L. infantum* JPCM5 predicted proteome. FDR < 0.05 was set as the threshold to define the functional enrichment significance.

**Fig. 4:**
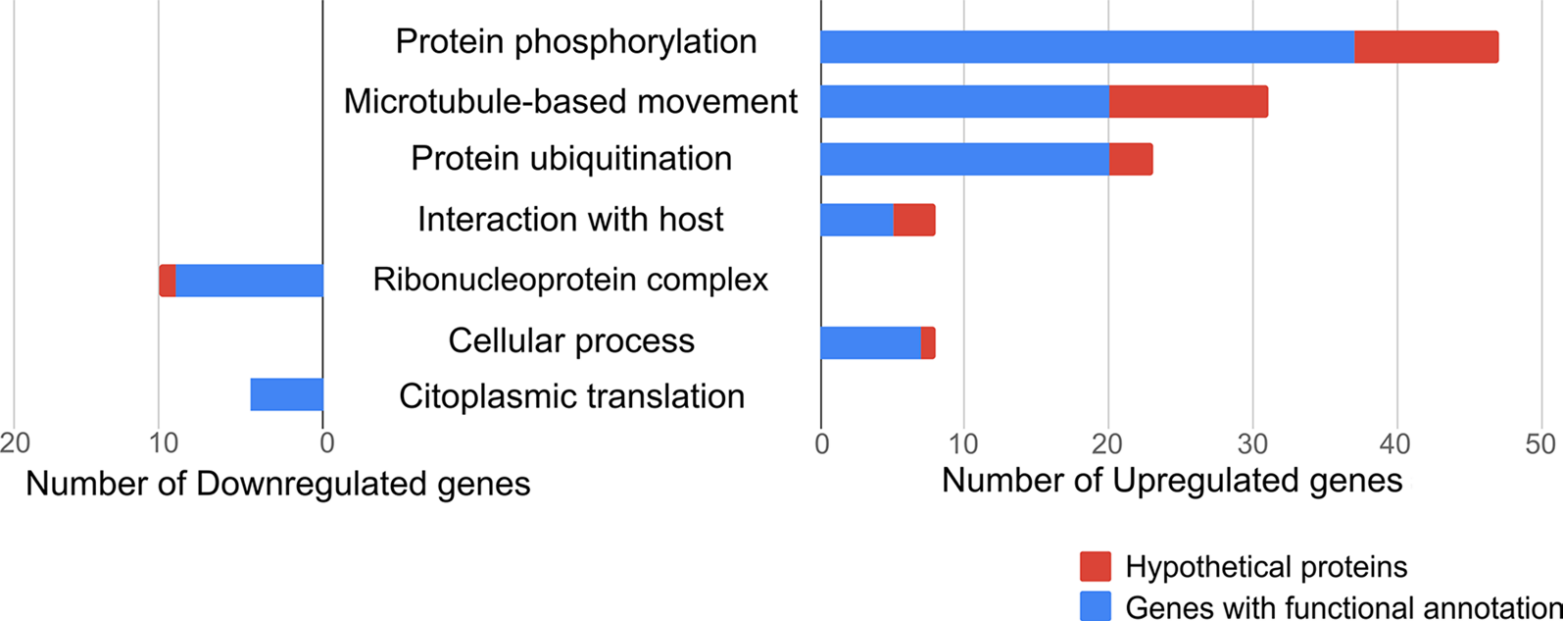
Differentially expressed (DE) genes for the most representative GO-enriched terms for the biological process category. The figure shows the most representative GO terms for the not enriched but differentially expressed (up- and downregulated) dataset. Blue bars represent the total number of genes with functional annotation for each term, and red bars represent the total number of hypothetical proteins for each category, in the same dataset

Gene ontology enrichment analysis of 837 transcripts upregulated in the Sb^III^-resistant *L. infantum* showed that the overrepresented terms were related to quorum sensing (GO:0009372, GO:0052097 and GO:0052106), host–parasite interaction (GO:0044764, GO:0044114, GO:0044115 and GO:0044145), protein modifications (GO:1903320 and GO:0032446), post-translational modifications, protein phosphorylation (GO:0006468) and protein ubiquitination (GO:0016567), microtubule-based movement (GO:0007018) and regulation of membrane lipid distribution (GO:0097035) (Figs. 2, 4).

In contrast, GO enrichment analysis of 96 transcripts downregulated in the Sb^III^-resistant *L. infantum* showed overrepresentation of all GO terms linked with rRNA processing (GO:0006364), nucleosome assembly (GO:0034622, GO:0022607, GO:0065003, GO:0022618, GO:0070925, GO:0042255, GO:0000028, GO:0006334, GO:0031497, GO:0006333, GO:0065004 and GO:0034063) and maintenance of translational fidelity (GO:1990145) (Figs. 3, 4).

### RNA profiling of *L. infantum* (MHOM/BR/74/PP75)

#### Genes upregulated in the LiSbR line

Out of 431 (431/837, 51.49%) upregulated enriched genes in the Sb^III^-resistant *L. infantum* line, 124 (124/431, 28.77%) presented GO ontology on the biological process (111 enriched and 13 without enrichment), 289 (289/431, 67.05%) genes did not present GO ontology on the biological process, and 18 (18/431, 4.18%) genes had not GO ontology associated (Additional file 1: Table S1, Additional file 2: Table S2 and Additional file 3: Table S3, respectively).

According to GO enrichment analysis, some terms related to biological processes were under- or overrepresented in the differentially expressed genes (Fig. 2). The most representative GO terms were protein phosphorylation, microtubule-based movement, protein ubiquitination, cellular process, quorum sensing involved in interaction with hosts and others (Fig. 4). Data from other DE genes up- and downregulated in the LiSbR line that presented GO enrichment are described on Tables 2 and 3. These data are representative of the complete results given in Additional file 1: Table S1.

**Table 2:** Upregulated enriched genes associated to the Gene Ontology biological process category

ID	Description	Fold change	*P*-value
Protein phosphorylation			
LINF_320013700	CYC2-like cyclin—putative^a^	5.56	3.34E−42
LINF_340027100	Dual specificity protein phosphatase—putative	8.93	4.34E−107
LINF_360054700	Elongation factor-2 kinase-like protein	3.23	2.10E−16
LINF_350046100	Kinetoplastid kinetochore protein 3—putative	2.02	9.69E−13
LINF_250012200	Myosin heavy chain kinase c-like protein	3.37	3.77E−20
LINF_300023300	Phosphatidylinositol kinase—putative^a^	3.97	4.10E−42
LINF_360034100	Protein kinase—putative^a^	3.88	5.44E−42
LINF_010011100	Rab-GTPase-TBC domain-containing protein	2.13	1.17E−16
LINF_340047100	Target of rapamycin kinase 3^a^	2.74	2.33E−21
Microtubule-based movement			
LINF_070010300	Dynein heavy chain^a^	5.90	6.51E−54
LINF_130011900	Kinesin—putative^a^	5.87	1.11E−70
LINF_330038800	Present in the outer mitochondrial membrane	4.06	1.74E−37
LINF_260024800	Microtubule-associated protein—putative	3.73	1.67E−59
LINF_270017700	Intraflagellar transport protein 88—putative	2.16	2.53E−19
LINF_040010500	Tetratricopeptide repeat	2.14	2.06E−19
LINF_210017400	WD40 repeat-containing protein	2.06	4.35E−21
Biosynthetic process			
LINF_350008300	Serine palmitoyltransferase—putative	2.99	2.23E−35
LINF_170011100	Phenazine biosynthesis-like protein	2.15	3.76E−08
Cellular process			
LINF_360020600	N-terminal region of chorein	4.95	3.27E−59
LINF_190015000	FYVE zinc finger-containing protein	3.66	2.95E−27
LINF_270009800	Glycosomal transporter (GAT3)—putative	3.39	1.48E−39
LINF_240020050	Multi-drug resistance protein-like	3.27	1.11E−33
LINF_350025400	Ankyrin repeat protein—putative	2.67	1.11E−21
Cellular component organization			
LINF_130020500	Phosphoprotein phosphatase—putative	2.28	1.71E−11
Protein ubiquitination			
LINF_350059500	SPRY domain; HECT-domain (ubiquitin-transferase)^a^	9.14	4.18E−81
LINF_360053000	Ubiquitin protein ligase—putative^a^	7.30	3.59E−111
LINF_220017000	Zinc finger—C3HC4 type (RING finger)	3.77	2.20E−40
LINF_110017600	Protein transport protein SEC31—putative	3.57	6.80E−54
LINF_160017100	WW domain-containing protein	2.63	5.06E−15
LINF_160018000	Cullin family	2.23	1.06E−15
Ribonucleoprotein complex assembly			
LINF_170016300	ATP-dependent RNA helicase—putative	2.38	1.49E−26
Stress granule assembly			
LINF_180019600	Pumilio protein 2—putative	5.59	6.98E−64
Cellular metabolic process			
LINF_270018400	RING-H2 zinc finger	3.33	9.20E−24
LINF_290018600	Heat shock protein 100 kDa	2.86	5.67E−32
Primary metabolic process; cellular macromolecule biosynthetic process; cellular nitrogen compound metabolic process			
LINF_300022800	DNAJ domain protein—putative	2.13	9.30E−14
Primary metabolic process; macromolecule metabolic process; nitrogen compound metabolic process			
LINF_120008300	Myotubularin-related protein—putative	4.00	4.38E−52
Multi-organism cellular process			
LINF_310038500	Acetyl-CoA carboxylase—putative	2.74	5.28E−33
Regulation of membrane lipid distribution; phospholipid translocation			
LINF_340032300	Phospholipid-transporting ATPase-like protein^a^	3.69	2.87E−39
Regulation of membrane lipid distribution			
LINF_260032600	ATP-binding cassette protein subfamily B—member 2—putative	2.21	7.28E−13
Quorum sensing involved in interaction with host; multi-organism cellular process; modulation of symbiont involved in interaction with host			
LINF_330022900	RNA recognition motif. (a.k.a. RRM–RBD or RNP domain)^a^	14.4	1.07E−153
LINF_330023000	RNA-binding protein—putative	4.01	1.25E−12

^a^Genes with more than one copy in the corresponding GO category. See Additional file 1: Table S1 for complete data

**Table 3:** Downregulated enriched genes associated to the Gene Ontology biological process category

ID	Description	Fold change	*P*-value
Nucleosome assembly			
LINF_100016800	Histone H3—putative	2.54	5.91E−28
LINF_170019500	Histone H2B^a^	2.34	4.29E−09
LINF_310040900	Histone H4	2.15	6.51E−18
Ribonucleoprotein complex subunit organization; ribosome biogenesis			
LINF_110013300	40S ribosomal protein S21—putative^a^	2.77	2.71E−31
LINF_070010600	60S ribosomal protein L7a—putative^a^	2.41	3.03E−17
LINF_010009200	Ribosomal protein S7—putative	2.36	9.19E−23
LINF_360008600	Nuclear protein family a—putative	2.11	4.18E−08
Translation			
LINF_290032300	60S ribosomal protein L13—putative^a^	2.19	2.28E−16
LINF_130017200	40S ribosomal protein S4—putative	2.02	8.88E−14
Ribosome biogenesis			
LINF_060009400	Ribosomal protein L19e—putative^a^	3.14	1.54E−35
LINF_350023700	60S ribosomal protein L5—putative	3.00	9.31E−33
LINF_360015100	40S ribosomal protein S18—putative	2.35	9.75E−17
Ribonucleoprotein complex subunit organization			
LINF_210018200	40S ribosomal protein S23—putative^a^	2.62	1.59E−24
LINF_030007300	Ribosomal protein L38—putative	2.24	1.49E−22
Ribonucleoprotein complex subunit organization; rRNA processing; ribosome biogenesis			
LINF_260021300	40S ribosomal protein S33—putative	2.66	1.95E−25
LINF_340050700	Nucleolar protein family a—putative	2.10	2.15E−09
Cytoplasmic translation			
LINF_330028300	60S ribosomal protein L37^a^	2.27	7.94E−20
LINF_350009300	40S ribosomal protein S3A—putative	2.00	3.20E−12

^a^Genes with more than one copy in the corresponding GO category. See Additional file 1: Table S1 for complete data

Thirty-seven transcripts belonging to the protein phosphorylation category were upregulated in the LiSbR line (Table 2, Additional file 1: Table S1 and Additional file 2: Table S2). This group includes five transcripts encoding phosphatidylinositol kinase (PIK) (2.52 to 3.97-fold upregulated); RAB GTPases (2-fold upregulated); dual specificity protein phosphatase (DUSP) (8.93-fold upregulated); protein phosphatase and protein phosphatase 2C (2.28 to 17.52-fold upregulated, respectively); cyclins 10, 11 and CYC2-like (2.27 to 5.56-fold upregulated) and elongation factor 2 (EF2) (3.23-fold upregulated).

In the microtubule-based movement category, 20 transcripts were upregulated in the LiSbR line (Table 2 and Additional file 1: Table S1), including dyneins (2.04 to 5.9-fold upregulated); ten transcripts encoding kinesins (2.02 to 5.87-fold upregulated); tryptophan-aspartic acid (WD) protein (2-fold upregulated) and tetratricopeptide repeat domain (TPR) (two-fold upregulated).

GO enrichment analysis also showed that transcripts related to protein ubiquitination were upregulated in the LiSbR line. Twenty transcripts (2.03 to 9.14-fold upregulated in the LiSbR line) were assigned for this category, such as ubiquitin, ubiquitin-transferase, cullin protein and zinc finger-containing proteins. Four transcripts encoding different zinc finger proteins (C3HC4 type—RING finger and FYVE) were 2.15 to 3.77-fold upregulated in the LiSbR line (Table 2 and Additional file 1: Table S1). Glycosomal transporter (GAT3) was 3.39-fold upregulated in the LiSbR line.

Other categories were also present among the upregulated transcripts. Serine palmitoyltransferase, included in the biosynthetic process category, was 2.99-fold upregulated in the LiSbR line (Table 2 and Additional file 1: Table S1). Transcripts encoding ATP-dependent RNA helicase were 2.38 to 3.34-fold upregulated in the LiSbR line (Table 2, Additional file 1: Table S1 and Additional file 2: Table S2) and were included in the ribonucleoprotein complex assembly category. In the stress granule assembly category, one transcript assigned as pumilio protein was 5.59-fold upregulated in the LiSbR line. Four transcripts related to phospholipid-transporting ATPase/P-type were upregulated in the LiSbR line. In the cellular metabolic process category, a transcript encoding a 100 kDa heat shock protein was 2.86-fold upregulated in the LiSbR line. In the categories primary metabolic process, cellular macromolecule biosynthetic process and cellular nitrogen compound metabolic process, DNAJ was found to be 2.13-fold upregulated in the LiSbR line. Many transcripts belonging to ATP-binding cassette (ABC) transporters were 2.2 to 4.6-fold upregulated in the LiSbR line and were included in the category regulation of membrane lipid distribution and phospholipid translocation. Among the transcripts implicated in quorum sensing involved in interaction with host and multi-organism cellular processes, four transcripts of the RNA recognition motif (RRM) were 2.87 to 14.4-fold upregulated in the LiSbR line.

#### Genes downregulated in the LiSbR line

Out of 73 (73/96, 76.01%) enriched transcripts downregulated in the Sb^III^-resistant *L. infantum* line, 37 (37/73, 50.68%) presented GO enrichment on biological process, six (6/73, 8.22%) genes did not present GO ontology on this category, and 30 (30/73, 41.09%) genes had no GO term associated (Additional file 1: Table S1, Additional file 2: Table S2 and Additional file 3: Table S3, respectively). According to GO enrichment analysis, some terms related to biological processes were under- or overrepresented in the DE genes (Fig. 3, Table 3 and Additional file 1: Table S1). The most representative GO terms were ribonucleoprotein complex subunit organization, rRNA processing, ribosome biogenesis, translation and nucleosome assembly.

According to GO enrichment analysis, a group of terms related to ribosomes were overrepresented in the DE dataset. The transcripts encoding ribosomal proteins such as ribosomal proteins 40S and 60S and nucleolar and nuclear proteins are downregulated in the LiSbR line.

Fourteen transcripts encoding ribosomal proteins of the small ribosomal 40S subunit, 40S ribosomal S3a, S4, S15, S16, S17, S18, S19, S21, S23 and S33 were 2.01 to 3.12-fold downregulated in the LiSbR line (Table 3). In addition, 11 transcripts encoding ribosomal protein components of the 60S subunit of the large ribosomal subunit—60S ribosomal L5, L7a, L11, L13, L18a, L21, L22, L31, L35 and L37—were 2.02 to 3.0-fold downregulated in the LiSbR line (Table 3).

Transcripts encoding the histones H2A, H2B, H3 and H4 were found 2.0 to 2.56-fold downregulated in antimony-resistant *L. infantum* line (Table 3, Additional file 1: Table S1 and Additional file 3: Table S3) and were included in the nucleosome assembly category.

### Proteins without GO enrichment for biological process

Some differentially expressed transcripts were not related to any category in the GO enrichment analysis (Additional file 2: Table S2), including, for example: 60S ribosomal L23a (2.07-fold upregulated in the LiSbR line); cytochrome b5 and cytochrome P450 reductase (6.37 and 4.33-fold upregulated in the LiSbR line, respectively); gamma-glutamylcysteine synthetase (2.6-fold upregulated in the LiSbR line); mannosyltransferase (2.54-fold upregulated in the LiSbR line) and two transcripts encoding protein classified as amastin (3.33- and 2.97-fold upregulated in the LiSbR).

### Hypothetical proteins

Data from comparative transcriptomic analysis of susceptible and Sb^III^-resistant *L. infantum* lines showed that 429 differentially expressed transcripts were assigned as hypothetical protein without predicted function. From these, 406 transcripts were upregulated and 23 were downregulated in the LiSbR line (Table 1). Out of 429 DE transcripts, 46 presented GO ontology on biological process (32 functionally enriched and 13 without enrichment), 142 transcripts did not present GO ontology on biological process, and 241 genes had no GO term associated (Additional file 4: Table S4). According to GO enrichment analysis, some terms related to biological processes were under- or overrepresented in the differentially expressed transcripts (Figs. 2, 3, 4). Similar to DE genes, the main terms enriched were microtubule-based movement, protein phosphorylation, protein ubiquitination, quorum sensing involved in interaction with host, ribonucleoprotein complex and others.

## Discussion

Chemotherapy against leishmaniasis remains the main strategy to manage disease control, but several implications regarding the treatment should be considered [11–17]. Pentavalent antimonials are considered one of the main options of treatment; however, these drugs have several toxic side effects and high resistance rates [9, 11–13, 16, 17]. Thus, the comprehension of resistance molecular mechanisms in *Leishmania* spp. is crucial to identify potential drug targets to prevent or reverse such mechanisms. In this study, RNA Seq has been used successfully to quantify transcript levels of Sb^III^-resistant and wild-type *L. infantum* lines. Our results showed that many pathways upregulated in the antimony-resistant *L. infantum* line are associated to signaling networks, such as kinases and phosphatases; microtubule-based movement, such as dyneins and kinesins; protein ubiquitination; stress response, such as HSP-100 and DNAJ; regulation of membrane lipid distribution, such as ATP-binding cassette; proteins associated to RNA metabolism, such as RNA-binding proteins, pumilio and other proteins involved in important metabolic pathways. Interestingly, our data revealed that the transcripts encoding ribosomal proteins such as 40S and 60S ribosomal proteins, nucleolar and nuclear proteins and histones are downregulated in the antimony-resistant *L. infantum* line. These results show downregulation of genes involved in translation and ribosome biogenesis then modulating important pathways associated with antimony resistance phenotype in *L. infantum*.

Some of the differentially expressed transcripts identified in this study corroborate previous proteomic analysis of antimony-resistant and -susceptible *L. infantum* lines [22]. In addition, some upregulated transcripts identified in this study were also previously investigated by our group, such as gamma-glutamylcysteine synthetase [41] elongation factor 2 [42] and mannosyltransferase [43], and these previous results confirmed that they are associated with antimony resistance phenotype in *Leishmania* spp.

An interesting category demonstrated to have differentially expressed transcripts was the “protein phosphorylation” category. Protein phosphorylation is one of the most important post-translational modifications regulating various signaling processes. GO enrichment analysis showed that 37 transcripts belonging to the protein phosphorylation category are 2.04 to 8.93-fold upregulated in the LiSbR line (Table 2 and Additional file 1: Table S1). Protein kinases are important regulators of many different cellular processes, such as transcriptional control, cell cycle progression, differentiation and response to stress [44, 45]. They represent promising drug targets for trypanosomes and *Leishmania*, since some of them are essential for viability of parasites and have significant sequence differences from mammalian homologues [44].

A comparative proteomic and phosphoproteomic analyses of Sb^III^-resistant and -susceptible lines of *L. braziliensis* identified several potential candidates for biochemical or signaling networks associated with antimony-resistance phenotype in this parasite [22, 23]. In the antimony-resistant *L. infantum* line, we also observed that different kinases and phosphatases are differentially expressed in this parasite (Additional file 1: Table S1 and Additional file 2: Table S2). RAB GTPases (whose transcripts were shown to be two-fold upregulated in the LiSbR) play a key role in regulation of exocytic and endocytic pathways in eukaryotic cells. This protein was also more abundant in the LiSbR line [22, 46]. It has been shown that RAB GTPases of *L. major* are highly immunogenic in individuals immune to cutaneous and visceral leishmaniasis [47]. *L. donovani* overexpressing RAB6 showed a resistant phenotype by allowing trans-dibenzalacetone-treated parasites to both increase internal thiol levels and enhance MRP pump activity [47].

Elongation factor 2 (EF2), a relevant factor for production of proteins, can be regulated through inhibitory phosphorylation at threonine 56 by EF2 kinase [48]. The transcripts of this enzyme were 3.23-fold upregulated in the LiSbR line. Our group showed that the EF2-overexpressing *L. braziliensis* clone was slightly more resistant to EF2K inhibitor than the WTS line. Surprisingly, this inhibitor increased the antileishmanial effect of Sb^III^, suggesting that this association might be a valuable strategy for leishmaniasis chemotherapy [22, 42].

Other transcripts associated with dephosphorylation, such as protein phosphatase and protein phosphatase 2C, were 17.52 to 2.28-fold upregulated in the LiSbR line, respectively (Additional file 1: Table S1 and Additional file 2: Table S2). Proteomic analysis using these same *L. infantum* lines showed that both enzymes were also more abundant in the Sb^III^-resistant line [22].

The category of “protein ubiquitination” was also a category whose transcripts were differentially expressed in the resistant parasites. Ubiquitination is a crucial process in all eukaryotic organisms. It is involved in several essential functions, such as degradation of denatured proteins, DNA repair, endocytosis, regulation of protein levels, transcription, and apoptosis [49]. Twenty transcripts that are 2.03 to 9.14-fold upregulated in the LiSbR line were assigned to this category, described as: ubiquitin, ubiquitin-transferase (HECT domain—homologous to the E6-AP carboxyl terminus and SPRY domain—SPla and the RYanodine receptor), cullin protein (involved in ubiquitination through participation in multisubunit ubiquitin ligase complexes), zinc finger-containing proteins and others (Table 2 and Additional file 1: Table S1). Similar to our results, antimony-resistant *L. tropica* isolate also showed overexpression of ubiquitin [50]. These data suggest that increased levels of protein ubiquitination may contribute to degradation of oxidized proteins, protecting the parasite against oxidative stress from antimony.

The zinc finger proteins, serine palmitoyltransferase and ATP-dependent RNA helicase, grouped respectively in the categories of “cellular process,” “biosynthetic process” and “ribonucleoprotein complex assembly,” also had their transcripts differentially expressed in the present work. Zinc finger proteins are RNA-binding proteins involved in many biological processes by binding nucleic acids or participating in transcriptional or translational processes by mediating protein-protein interactions and membrane association [51]. Zinc finger domains in proteins were recently proposed as potential targets for Sb^III^ because of the ability of Sb^III^ to compete with Zn^II^. A previous study suggested that the interaction of Sb^III^ with zinc finger proteins may modulate the pharmacological action of antimonial drugs [10, 14, 52]. In our study, four transcripts encoding different zinc finger proteins (C3HC4 type—RING finger and FYVE) were 2.15 to 3.77-fold upregulated in the LiSbR line line (Table 2 and Additional file 1: Table S1). The FYVE domain is a small zinc-binding module that recognizes phosphatidylinositol 3-phosphate, and the majority of these proteins are implicated in membrane trafficking, protein sorting and signaling transduction [53].

Serine palmitoyltransferase catalyzes the first rate-limiting step in the synthetic pathway of *de novo* sphingolipid biosynthesis [54]. This enzyme was 2.99-fold upregulated in the LiSbR line line (Table 2 and Additional file 1: Table S1). Metabolomic analysis revealed differences in the phospholipid and sphingolipid contents between antimony-susceptible and -resistant *L. donovani* isolates [55]. According to Zhang and Beverley [56], these two lipid classes are both abundant and critical to virulence and viability in *Leishmania.*

Transcripts encoding ATP-dependent RNA helicase were 2.38 to 3.34-fold upregulated in the LiSbR line (Table 2, Additional file 1: Table S1 and Additional file 2: Table S2). It plays an essential function in RNA metabolism, including RNA degradation, translation, regulation and RNA editing [57, 58]. A member of RNA helicases “DDX3 DEAD-Box” of *Leishmania* have been shown to play a central role in preventing reactive oxygen species-mediated damage and in maintaining mitochondrial protein quality control [58].

Interestingly, four transcripts related to phospholipid-transporting ATPase/P-type ATPase were upregulated in the LiSbR line. These transcripts were grouped into the category “regulation of membrane lipid distribution; phospholipid-translocating.” ATPases are membrane proteins that perform active ion transport across biological membranes, which are found in bacteria and all eukaryotic cells, including *Leishmania* [59]. Fernandez-Prada et al. [60] demonstrated that different point mutations in a P-type ATPase transporter in *L. infantum* are implicated with cross-resistance to miltefosine and amphotericin B.

The categories of “cellular metabolic process” and “primary metabolic process; cellular macromolecule biosynthetic process; cellular nitrogen compound metabolic process” also showed transcripts differentially expressed in parasites resistant to Sb^III^ such as HSPs and DNAJ proteins. The HSPs have important functions in folding, secretion, assembly, intracellular localization, regulation and degradation of other proteins [61]. In general, the heat shock response is a homeostatic mechanism that protects cells from the deleterious effects of environmental stress, such as heat and drug exposure [62]. Several authors reported the overexpression of HSPs in antimony-resistant isolates of *L. donovani* [63–65], *L. braziliensis* and *L. infantum* lines [22, 66]. Here, a transcript encoding a 100 KDa heat shock protein was 2.86-fold upregulated in the LiSbR line. HSP100 has the unique capability of recognizing misfolded proteins within an aggregate and actively unfolding them, ultimately disassembling the insoluble structure and delivering substrates into refolding pathways [67].

DNAJ proteins, also known as HSP40s, are crucial partners for HSP70 chaperones, and much of the functional diversity of the HSP70s is driven by this diverse class of cofactors [67]. Here, DNAJ was 2.13-fold upregulated in the LiSbR line. This protein plays a relevant role in the differentiation process from the promastigote to amastigote stage in *L. infantum*, since it suffers a dramatic increase in phosphorylation [68]. Interestingly, HSP40 was also found increased in artemisinin-resistant *L. donovani* [69].

Interestingly, in our study many transcripts belonging to ATP-binding cassette (ABC) transporters were upregulated in the LiSbR line. These transcripts were grouped in the category of “regulation of membrane lipid distribution; phospholipid translocation.” ABC transporters comprise a superfamily of integral membrane proteins involved in the ATP-dependent transport of a variety of molecules across biological membranes, including amino acids, sugars, peptides, lipids, ions and chemotherapeutic drugs [70]. They have been associated with drug resistance in various diseases. In *Leishmania*, the first ABC protein identified was MRPA (multidrug resistance protein, PgpA) [71] which is a member of the ABCC subfamily, able to confer resistance to antimonials by sequestering thiol-metal conjugates into an intracellular vesicle [71, 72]. Our previous data showed an association of chromosomal amplification of *MRPA* gene with the drug resistance phenotype in Sb^III^-resistant *Leishmania* spp. lines [22, 72]. Similarly, it has been shown that *L. infantum* knockout for the *MRPA* gene is more sensitive to Sb [73]. As ABC transporters are important regulators of drug susceptibility, they are excellent candidates for inhibitor design [74].

Since the regulation of gene expression in trypanosomatids occurs largely at post-transcriptional levels, the main control points in gene expression are mRNA degradation and translation [75]. The RNA-binding proteins (RBP) play essential roles in regulating RNA processing, transport, localization, translation and degradation. RBPs contain various structural motifs, such as RNA recognition motif (RRM), dsRNA-binding domain, zinc finger and others [76]. Four transcripts of RRM were 2.87 to 14.4-fold upregulated in the LiSbR line.

Other transcripts differentially expressed in parasites resistant to Sb^III^ could not be classified in any GO enrichment for biological processes, such as ribosomal proteins, cytochrome b5, cytochrome P450 reductase, gamma-glutamylcysteine synthetase and mannosyltransferase. Ribosomal proteins play an important role in the translation, and they also regulate cell growth and apoptosis. In our study, the 60S ribosomal L23a, a component of the 60S subunit of the ribosome large subunit, was found 2.07-fold upregulated in the LiSbR line (Additional file 2: Table S2). In agreement with our results, proteomic analysis showed that this protein also was overexpressed in antimony-resistant *L. donovani* isolates [77]. Interestingly, 60S ribosomal L23a-overexpressing *L. donovani* line was more resistant to sodium antimony gluconate (Sb^V^), miltefosine and paromomycin [78].

Cytochrome b5 and cytochrome P450 reductase, which are involved in oxidoreductase activity, were 6.37- and 4.33-fold upregulated in the LiSbR line, respectively (Additional file 2: Table S2). Cytochrome b5 is a flavohemoprotein associated with oxidative reactions such as catabolism of xenobiotics and compounds of endogenous metabolism [79]. Mukherjee et al. [80] observed that *L. major* deficient in cytochrome b5 oxidoreductase domain presents decreasing of linoleate synthesis followed by increased oxidative stress and cell death by apoptosis. Cytochrome P450 reductase is located on the endoplasmic reticulum in many types of cells and is also related to drug metabolism [80].

Gamma-glutamylcysteine synthetase (γ-GCS) is the first enzyme of the glutathione pathway that produces γ-glutamylcysteine, a direct precursor of glutathione [81]. Our results showed that one transcript encoding this enzyme was found 2.6-fold upregulated in the LiSbR line (Additional file 2: Table S2). γ-GCS has been shown to be essential for *L. infantum*, where it confers protection against oxidative stress and Sb^V^ [81]. An increase of GSH1 mRNA levels also has been reported in some *L. tarentolae* samples with in vitro-induced resistance to antimony [82] and some Sb^V^-resistant *L. donovani* field isolates [83]. Overexpression of γ-GCS is associated with antimony resistance phenotype in *L. guyanensis* [41].

Glycosylphosphatidylinositol is a surface molecule important for host–parasite interactions. Mannosyltransferase (GPI-14) is an essential enzyme for adding mannose on the glycosylphosphatidyl group. Our data showed that one transcript encoding this enzyme is 2.54-fold upregulated in the LiSbR line (Additional file 2: Table S2). Interestingly, our group overexpressed the *GPI-14* gene in *L. braziliensis* and observed the involvement of the GPI-14 enzyme in the Sb^III^ resistance phenotype of *L. braziliensis* [43].

## Conclusions

Transcriptomic profiling represents an important technology for analyzing the global changes in gene expression and regulation of the main metabolic pathways. This study allowed us to compare the transcriptome data from Sb^III^-resistant and wild-type *L. infantum* lines and identify a robust set of transcripts from several biochemical pathways that are associated with the antimony resistance phenotype in this parasite. Overall, our results support the idea that the antimony resistance mechanism in *Leishmania*, similar to other organisms, is complex and multifactorial. The proteins encoded by DE genes may be further evaluated as molecular targets for new drugs against leishmaniasis. In addition, functional studies will be performed to determine the role of some hypothetical proteins and genes with unknown function in the antimony resistance phenotype in *Leishmania*.

## Supplementary information

**Additional file 1: Table S1.** Enriched genes for biological process category with Gene Ontology assigned terms.

**Additional file 2: Table S2.** Not enriched genes for the biological process category with Gene Ontology assigned terms.

**Additional file 3: Figure S1.** Not enriched genes for the biological process category without Gene Ontology assigned terms.

**Additional file 4: Table S3.** Hypothetical proteins: enriched for the biological process (BP) category, not enriched for BP with and without Gene Ontology assigned terms.

## Data Availability

The datasets supporting the conclusions of this article are included within the article and its additional files. Sequences generated during the present study were deposited at NCBI database under SRA accession numbers SRX2833233, SRX2833324, SRX2833326, SRX2833322, SRX2833327. BioSample accession numbers SAMN07137473, SAMN07137475 and BioProject accession number PRJNA348689.

## Abbreviations

ABC transporter: ATP-binding cassette transporter
DE: Differentially expressed
EC_50_: Effective concentration required to decrease growth by 50%
FC: Fold change
GFF: General feature format
GO: Gene ontology
RIN: RNA integrity number
SAM: Sequence alignment map
Sb^V^: Pentavalent antimony
Sb^III^: Trivalent antimony
VL: Visceral leishmaniasis
